# Plasmalogen Profiling in Porcine Brain Tissues by LC-MS/MS

**DOI:** 10.3390/foods12162990

**Published:** 2023-08-08

**Authors:** Yue Wu, Yifan Chen, Min Zhang, Hitoshi Chiba, Shu-Ping Hui

**Affiliations:** 1 Faculty of Health Sciences, Hokkaido University, Kita-12, Nishi-5, Kita-ku, Sapporo 060-0812, Japan; wuyue.hokudai@gmail.com (Y.W.); ovd61363@elms.hokudai.ac.jp (Y.C.); 2 GLB Co., Ltd., 2-8 Mikage 2 Chome, Higashinada-ku, Kobe 658-0047, Japan; zhangmin@kobe.zaq.jp; 3 Department of Nutrition, Sapporo University of Health Sciences, Nakanuma Nishi-4-2-1-15, Higashi, Sapporo 007-0894, Japan; chiba-h@sapporo-hokeniryou-u.ac.jp

**Keywords:** porcine brain, porcine brain-derived glycerophospholipids, plasmalogen, dietary phospholipids, molecular species, fatty acyl, nutritional value, LC-MS/MS

## Abstract

Plasmalogen, a functional glycerophospholipid, is known for its beneficial nutritional effects, such as anti-oxidation and anti-inflammation. As the porcine brain is a plasmalogen-rich resource, this study aimed to explore its potential for plasmalogen-based health food product development, with special attention on whether and how the industrial production processes influence the plasmalogen content and composition. In the present work, plasmalogens from different porcine brain products were investigated using liquid chromatography–tandem mass spectrometry. The results indicated that all the porcine brain products showed abundant total plasmalogens, of which more than 95% were ethanolamine plasmalogen species. Acetone precipitation, ethanol extraction, and drying did not significantly affect the plasmalogen content, whereas repeated freeze-thaw cycles in the production process led to noticeable loss. The chemometric investigation suggested that raw products and glycerophospholipid products exhibited different profiles; furthermore, the concentration step seemed to impact the plasmalogen composition. The nutritional assessment revealed that porcine brain products showed favorable values of multiple indexes, including PUFA/SFA ratio, n-6/n-3 ratio, thrombogenicity index, and unsaturation index, suggesting a health-beneficial value. The current study not only shows the feasibility of producing porcine brain-derived plasmalogens, but also provides possible strategies for developing and quality-controlling dietary plasmalogen supplements and healthcare products.

## 1. Introduction

Plasmalogens are a particular subclass of glycerophospholipids, which have one fatty alcohol with a vinyl-ether bond at the sn-1 position and one fatty acyl at the sn-2 position of the glycerol backbone. Due to the headgroup at the sn-3 position, plasmalogens can be divided into ethanolamine plasmalogen (PlsEtn) and choline plasmalogen (PlsCho) as the primary two types [1,2]. Plasmalogens are proven to prevent oxidative damage [3,4], maintain mitochondrial function [5], inhibit inflammation [6], and suppress neuronal apoptosis in nerve cells [7,8], which could improve cognitive functions of Alzheimer’s disease patients and attenuate memory loss [9,10,11]. Our previous study also demonstrated that plasmalogen could inhibit the generation of ROS and reduce the oxidization and degradation of phospholipids by activating Nrf-2-dependent antioxidant enzymes [4]. Thus, plasmalogens are increasingly regarded as indispensable food bioactive compounds for human health.

It is fundamental to know the natural source of plasmalogens for their therapeutic studies and health product development. Animal foodstuffs, such as livestock, poultry, and seafood, are abundant with plasmalogens [2,12]. Therefore, plasmalogen-enriched extracts from animal sources are considered promising nutritional supplements. In most mammalian animals, plasmalogens are present tissues and the highest amount of plasmalogens is found in the brain, accounting for 22% of the total phospholipid, followed by the heart and the kidney [2].

Pig consumption has increased along with the population, during which a series of edible by-products are created, such as viscera, blood, and brains [13]. As an important derivation of pork processing, the porcine brain is consumed as a cuisine in some countries (e.g., “*Aeb Ong Aw*” in Thai and “*Naohua*” in China); moreover, it is also used as a traditional Chinese medicine, “*Zhunao*”. Nevertheless, consuming porcine brains is not common worldwide due to dietary habits, living customs, and religious restrictions. The porcine brain is an excellent source of lipids (e.g., phospholipids and fatty acids), as well as minerals (e.g., K, Na, Ca, and Mg) and proteins [14]. However, its high cholesterol content makes it risky. In 100 g (fresh weight) of porcine brain tissue, there is as high as 300 mg of cholesterol [14], which reaches the maximum limit of the recommended intake amount for an adult per day [15]. Therefore, it is necessary to find a healthier way to utilize the porcine brain, enriching the healthy ingredients, for instance, functional phospholipids. In particular, plasmalogens derived from the porcine brain draw our attention, and this has not been studied yet. The feasibility of extracting plasmalogens from the porcine brain tissue should be investigated to boost its nutritional value. It is noted that the processing of plasmalogen-contained foodstuffs may cause plasmalogen content loss together with composition alterations of its molecular species. However, the impacts of industrial production processes on plasmalogens in food products remain unknown.

In our previous studies, a reliable analytical approach for plasmalogens, including both PlsEtn and PlsCho species, using LC-MS/MS has been established and applied [16,17]. Being the subsequent research, the present study aimed to evaluate the plasmalogen content and composition in products derived from the porcine brain. We would like to assess the feasibility of extracting plasmalogens from porcine brain products and evaluate their characteristics. Moreover, for the porcine brain products produced using different process flows, their nutritional values, with regard to plasmalogens, were assessed. Such results are useful to establish a scientific basis for the development of plasmalogen-based dietary supplements.

## 2. Materials and Methods

### 2.1. Samples

**Scheme 1:**
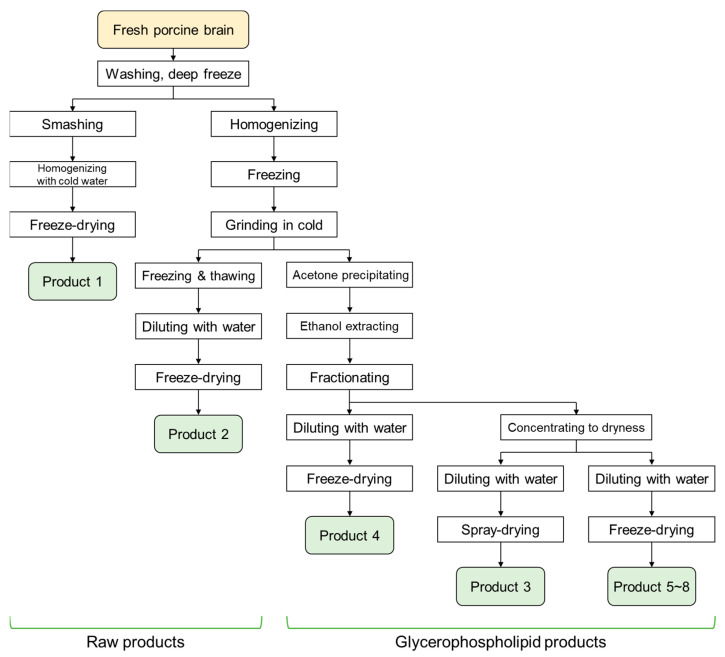
The workflow of producing the porcine brain products, including the raw products (No. 1 and No. 2) and the glycerophospholipid products (No. 3–No. 8).

All the porcine brain products were provided by Hebei Zhitong Bio-Pharmaceutical Co. Ltd. (Hebei, China). In this experiment, different porcine brain products were investigated, including the raw products and the porcine brain-derived glycerophospholipid products. Initially, the fresh porcine brain was washed three times with clean water and frozen at −80 °C. Different products were produced following the following workflow: (Scheme 1): (1) the frozen tissue was roughly smashed, homogenized with cold water, and then freeze-dried to obtain the No. 1 product. (2) The frozen tissue was homogenized and frozen again; next, the sample was ground in cold to become fine powder. The powder was diluted with water after being frozen and thawed, followed by freeze-drying to become the No. 2 product. (3) The ground sample was precipitated with acetone to remove the interfering fats and extracted with ethanol. The ethanol extract was fractionated to remove residues and diluted with water and freeze-dried to yield the No. 4 product. (4) The ethanol extract was concentrated to dryness, either diluted with water and spray-dried to obtain the product No. 3 or diluted with water and freeze-dried to obtain the products No. 5–No. 8.

For other tested samples, the egg-derived lecithin product (No. 9) and the soy-derived lecithin products (No. 10–No. 12) were commercially available and purchased from local markets in Japan. Detailed information of all the samples (No. 1–No. 12) is listed in Table S1.

### 2.2. Chemicals and Reagents for Analyzing

LC/MS grade solvents, including chloroform, methanol, water, and isopropanol, were obtained from Kanto Chemical Co., Inc. (Tokyo, Japan). The mobile phase additive ammonium acetate and the antioxidant butylated hydroxytoluene (BHT) were of analytical grade and obtained from Sigma-Aldrich (St. Louis, MO, USA). The two plasmalogen internal standards, PlsCho-p16:0/17:0 and PlsEtn-p16:0/17:0, were previously prepared by chemical synthesis in our laboratory [17]. Other chemicals were of analytical grade and obtained from Wako Pure Chemical Industries, Ltd. (Osaka, Japan) unless otherwise specified.

### 2.3. Sample Preparation for Analyzing

The sample (powder, c.a. 5 mg) was dissolved in cold chloroform/methanol 2:1 (*v*/*v*, with 0.002% BHT and 0.2 nmol of each internal standard), extracted by vortex, and dried under vacuum, as described previously [16,17]. After dryness, the extracts were dissolved in methanol, centrifugated to remove any insoluble, and transferred into glass vials for injection. For avoiding lipid auto-oxidation and degradation, the sample preparation was performed within 1 h. All samples were prepared in triplicate.

### 2.4. LC-MS/MS Analysis

A Prominence HPLC (Shimadzu Corp., Kyoto, Japan) coupled with an LTQ Orbitrap high-resolution mass spectrometer (Thermo-Fisher Scientific Inc., Waltham, MA, USA) was used in this experiment.

For chromatographic separation, an Atlantis T3 C18 column (2.1 mm × 150 mm, 3 µm; Waters Corp., Milford, MA, USA) was used with the temperature kept at 40 °C. The mobile phase consisted of 10 mM aqueous ammonium acetate (A), isopropanol (B), and methanol (C). The flow rate was set at 200 µL/min, and the gradient elution was programmed as below: 0.0–1.0 min, 40% A, 20% B, 40% C; 1.0–5.0 min, 20% A, 50% B, 30% C; 5.0–12.0 min, 5% A, 70% B, 25% C; 12.0–28.0 min, 3% A, 82% B, 15% C; 28.0–36.5 min, 3% A, 85% B, 12% C; 36.5–37.5 min, 40% A, 20% B, 40% C. The injection volume for each sample was 10 μL, and for minimizing the running time-induced variation, the injection order of the samples (the sequence) was randomized.

For MS detection, we used ESI-negative mode to detect both PlsEtn and PlsCho species. The spray voltage was set at 3.0 kV, and the capillary temperature was set at 330 °C; the sheath gas (N_2_) pressure and the auxiliary gas pressure (N_2_) were set at 50 psi and 5 psi, respectively. MS1 data were acquired by high-resolution full-scan, with a scan range of *m*/*z* 650–900 and a resolving power of 60,000. MS2 data were acquired by collision-induced dissociation and run in data-dependent mode, with a normalized collision energy of 35.0. The obtained raw data were processed by Xcalibur 2.3 (Thermo-Fisher Scientific Inc.). The semi-quantitation of plasmalogens was based on the peak area, according to the equation below:

(1) $${Amount}_{analyte}=\frac{{Peak\;area}_{analyte}}{{Peak\;area}_{internal\;standard}}\times {Amount}_{internal\;standard}$$

The plasmalogen species identified were annotated as “lipid class + total carbon number in the fatty chains (CN) + total double bond number in the fatty chains (DB)”. The calculated amount of each plasmalogen species was used for further data analyses: total plasmalogen, sum of all the detected plasmalogen species; total PlsEtn, sum of all the detected PlsEtn species; total PlsCho, sum of all the detected PlsCho species; headgroups and fatty chains were also calculated based on the amount of the responded species.

### 2.5. Statistical Analysis and Nutritional Assessment

Data were expressed as the means ± standard deviations (SD). Hierarchical cluster analysis (HCA) was performed using JMP^®^ 16 pro (SAS Institute Inc., Cary, NC, USA) with Ward’s linkage method, and principal component analysis (PCA) was performed using JMP without scaling or centering.

Nutritional assessment was based on the fatty acyls in plasmalogens. The following indexes were calculated: total saturated fatty acyls (Σ_SFA_), total monounsaturated fatty acyls (Σ_MUFA_), total polyunsaturated fatty acyls (Σ_PUFA_), PUFA/SFA ratio, n-6/n-3 ratio, thrombogenicity index (TI), unsaturation index (UI), and the sum of eicosapentaenoic acid and docosahexaenoic acid (EPA + DHA). Among them, the PUFA/SFA ratio, one of the most commonly used indexes, is widely used to evaluate the nutritional value of dietary foods, especially for assessing the impact of diet on cardiovascular health [18], whereas TI is considered to be useful to evaluate the degree of thrombogenicity in many studies concerning FA composition [19], which is calculated using the formula below:

(2) $$TI=\frac{C14:0+C16:0+C18:0}{\left(0.5\times \Sigma_{MUFA}\right)+\left(0.5\times \Sigma_{n-6\;PUFA}\right)+\left(3\times \Sigma_{n-3\;PUFA}\right)+(\frac{n-3\;PUFA}{n-6\;PUFA})}$$

whereas UI is used for indicating the unsaturation in lipids degree [20], which is calculated using the formula below:

(3) $$\begin{array}{l} UI=1\times \left(\Sigma_{monoenoics\%}\right)+2\times \left(\Sigma_{dienoics\%}\right)+3\times \left(\Sigma_{trienoics\%}\right)\\ \;\;\;\;+4\times \left(\Sigma_{tetraenoics\%}\right)+5\times \left(\Sigma_{pentaenoics\%}\right)\\ \;\;\;\;+6\times \left(\Sigma_{hexaenoics\%}\right) \end{array}$$

## 3. Results and Discussion

### 3.1. Characteristics and Residual Solvents of the Porcine Brain Products

All products were a pale-yellow powder with an egg-yolk-like flavor. In pursuit of confirming food safety and eliminating toxicity, tests were conducted to assess the residuals of organic solvents (namely, acetone and ethanol) utilized during the extraction process of the samples. The acetone residue among all samples was ≤0.003% and the ethanol residue under 0.005% (Table 1), both of which were in compliance with Pharmaceuticals for Human Use (ICH) regulations (Guidelines for Residual Solvents for Pharmaceutical Products) [21].

### 3.2. LC-MS/MS Analysis and Identification of Plasmalogen Species

**Figure 1:**
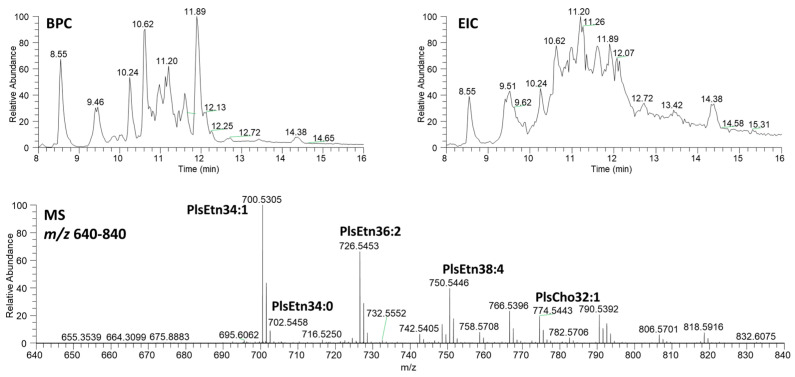
The representative base peak chromatogram (BPC) and extracted ion chromatogram (EIC) and MS1 spectrum of plasmalogen species of the porcine brain tissue samples.

In our previous study, we established a quantitative method for plasmalogen determination in various foodstuffs, which was proven to be highly selective and instrument-friendly [12,17]. Using HR-MS together with tandem MS, the representative LC/MS base peak chromatogram (BPC), extracted ion chromatogram (EIC), and the MS spectrum of the porcine brain tissue sample under negative modes are shown in Figure 1.

**Figure 2:**
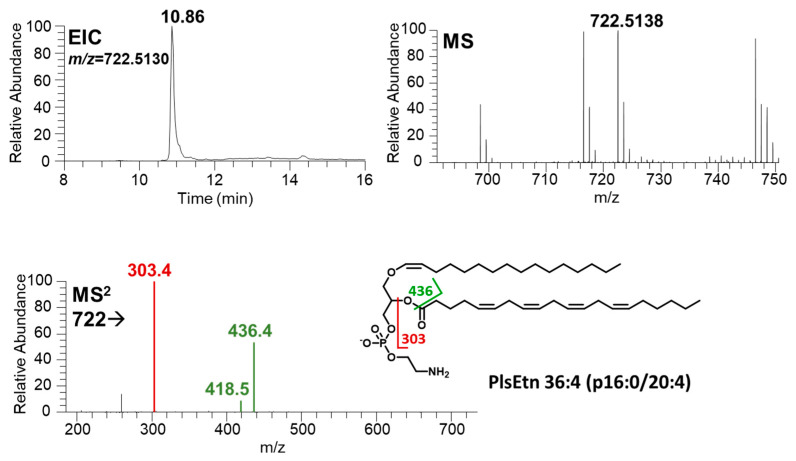
Identification of plasmalogen species in porcine brain tissue samples using high-resolution Orbitrap MS and tandem MS, taking PlsEtn 34:2 as an example.

Moreover, for the fatty acyl identification, taking PlsEtn 36:4 as an example shown in Figure 2, the major fragments in MS/MS spectrum were *m*/*z* 436, 418, and 303, indicating the loss of sn-2 acyl chain as ketene or acid and the sn-2 RCOO^−^ ion [22]; therefore, the fatty chain combination could be confirmed as p16:0/20:4. Based on their retention behavior on a reversed-phase column and the [M−H]^−^/[M+CH_3_COO]^−^ ion peaks on HRMS (compared with the authentic standards synthesized in our laboratory previously [4,12,17,23]) in these porcine brain tissue samples, a total of 26 plasmalogen species, including 22 PlsEtn and 4 PlsCho, were detected and identified (listed in Table 2). Such results were consistent with the literature that PlsEtn is the predominant plasmalogen in animal brains, accounting for approximately 90% [2,24].

In our preliminary research on the analysis of plasmalogen by tandem MS, we discovered challenges in identifying PlsCho isomers in positive ionization mode. In MS2, the major fragment observed was the headgroup (*m*/*z* 184), which made it difficult to distinguish different combinations of sn-1 fatty chains and sn-2 acyl chains, whereas in negative ionization mode, the major signals appeared from the fragmentation at the sn-2 position, thus enabling the identification of isomers [17]. Therefore, the negative ionization mode was primarily employed for plasmalogen analysis in our related research [16,17]. Moreover, although it was difficult to distinguish between plasmanyl and plasmenyl lipids using HR-MS or tandem MS, they could be separated using reversed-phase LC, according to Koch et al. [25]. Thus, the combination of RP-HPLC and tandem MS enabled reliable PlsCho and PlsEtn detection and further investigations. In addition, the consistency and reliability of the chromatographic results were confirmed. Yet, the resolution of the isomers PlsEtn36:3 (P-16:0/20:3 and P-18:1/18:2), PlsEtn38:4 (P-18:0/20:4 and P-16:0/22:4), and PlsEtn38:6 (P-18:2/20:4 and P-16:0/22:6) needed to be improved with the help of advanced techniques, such as ultra-performance LC or ion mobility spectrometry.

### 3.3. Comparison of Plasmalogen Amount

**Figure 3:**
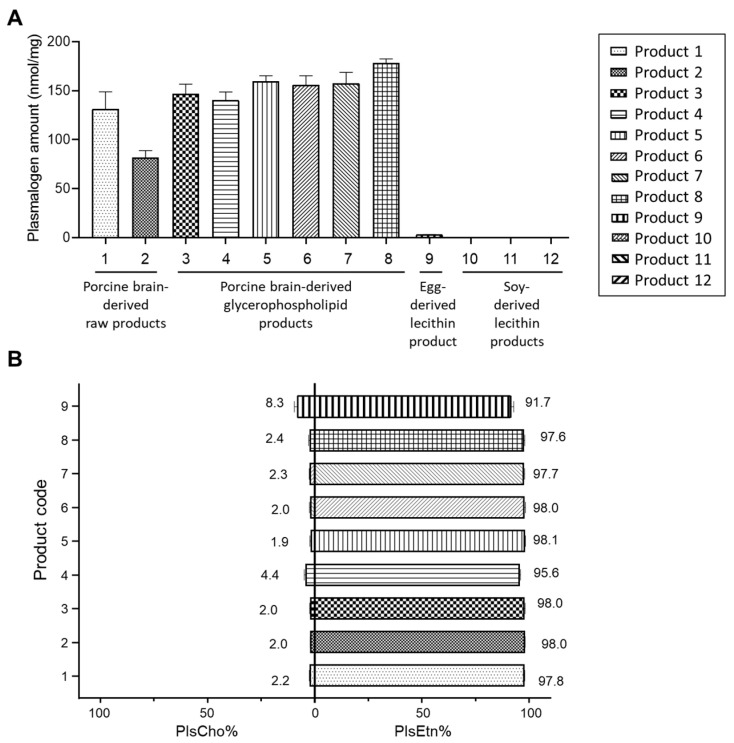
(**A**) Comparison of total plasmalogen amount. Data are expressed as means ± SD. (**B**) The proportion of ethanolamine and choline as the plasmalogen headgroup. Data are normalized as percentages and shown as means ± SD.

The total plasmalogen was defined as the sum of all the plasmalogen species, and the total plasmalogen amount was compared in 12 batches of samples, shown in Figure 3A. The plasmalogen content in the two kinds of raw product was 131.3 ± 17.6 nmol/mg (Product 1) and 81.7 ± 7.3 nmol/mg (Product 2), respectively. The plasmalogen content of Product 2 was nearly half that of Product 1, potentially influenced by the repeated freeze-thaw cycles in the preparation process, which might affect the plasmalogen content. In our previous research on the stability of plasmalogens in food, we confirmed that plasmalogens in meat products could be degraded and oxidized due to repeated freeze-thaw cycles [16]. This effect might be even more pronounced in food industry production. Therefore, the results of this experiment suggested minimizing freeze-thaw steps as much as possible during the preparation of food-derived plasmalogens to enhance their yield.

In the porcine brain-derived glycerophospholipid products, the plasmalogen content ranged from 140.2 ± 8.6 nmol/mg to 178.1 ± 4.3 nmol/mg. The steps of acetone precipitation for impurity removal and ethanol extraction do not result in a significant loss of plasmalogen content. Furthermore, for both the directly freeze-dried samples and the concentrated freeze-dried samples, no significant difference was observed in the plasmalogen content of the total plasmalogen fraction extracted using ethanol. Additionally, the drying method employed in the final step of producing the powdered product, whether it be relatively high-temperature spray-drying or relatively low-temperature freeze-drying, does not yield any variation in plasmalogen production. These findings indicate that despite the literature reporting the instability of plasmalogens due to their vinyl ether structure [2], the aforementioned factors do not have a significant negative impact on plasmalogen yield during the production of porcine brain plasmalogen products, as described in this study; thus, producers can focus their research efforts on aspects such as production costs and efficiency.

In addition, we found no plasmalogen detected in soy-derived lecithin products; it is indicated that the glycerophospholipids from animal sources were enriched with plasmalogen compared to those from plant sources, meaning more animal-derived glycerophospholipids could get a better nutrient supplement for our body needs. Moreover, the plasmalogen content in the egg-derived lecithin product only reached 3.1 ± 0.1 nmol/mg. It might be caused by different manufacturing processes; the inappropriate protocol may result in a considerable loss of the functional phospholipid plasmalogen.

Additionally, we comparatively investigated the phosphate headgroup of the plasmalogen species among the tested samples (Figure 3B). As a result, all the samples showed a more significant proportion of PlsEtn. The PlsEtn accounted for about 97.8% and 98.0% of the raw porcine brain tissue samples, respectively. For the porcine brain-derived glycerophospholipid products, the proportion of PlsEtn also showed a high ratio, excessing 95%, whereas for the egg-derived lecithin product, the PlsEtn proportion expressed a lower level, accounting for 91.7%. Although PlsEtn drew more interest in most previous studies due to its clinical significance and beneficial bioactivities [7,26], in our recent work, the other type of plasmalogen, PlsCho, also exhibited a comparable effect on human hepatocyte protection and oxidative damage attenuation [4]. Despite the fact that the distinctions of bioactive potentials between PlsEtn and PlsCho remain uncovered, our current findings could help provide an informative basis for plasmalogen sources targeting the headgroup (plasmalogen type) composition.

### 3.4. Characteristics of Plasmalogen Profile

**Figure 4:**
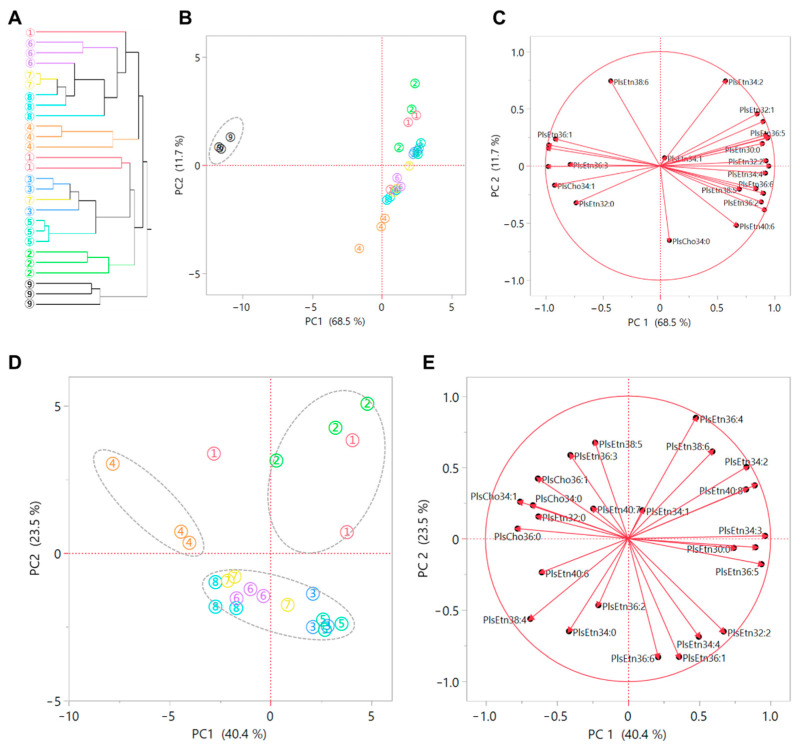
Multivariate statistics of the plasmalogen-contained dietary phospholipid samples. (**A**) Hierarchical cluster analysis (HCA); (**B**) PCA score plots of plasmalogen profiles; (**C**) PCA loading plots of plasmalogen profiles; (**D**) PCA score plots of plasmalogen profiles among all the porcine brain products; (**E**) PCA loading plots of plasmalogen profiles among all the porcine brain products. ①, product 1; ②, product 2; ③, product 3; ④, product 4; ⑤, product 5; ⑥, product 6; ⑦, product 7; ⑧, product 8; ⑨, product 9.

Besides the amount, plasmalogen species composition should also be considered in supplement and health care product development. Unsupervised HCA and PCA using the plasmalogen species variables were performed for all the samples (except Product 10–12, which did not contain plasmalogen), and the calculated results are shown in Figure 4. HCA revealed the diversity of the plasmalogen profiles in different samples (Figure 4A). In terms of PCA (Figure 4B,C), the first principal component (PC1) and the second principal component (PC2) accounted for 68.5% and 11.7% of the total variation, respectively (PC1 + PC2: 80.2%), which was considered to explain most of the variance adequately [27]. Both HCA and PCA revealed the distinctive molecular species characteristics between Product 9 (egg-derived lecithin product) and the other samples (i.e., porcine brain products). Moreover, taking a closer look at the loading plot, within the variables orientated towards the positive *x*-axis, there were more plasmalogen species with saturated fatty acyls, such as PlsEtn 36:1, PlsEtn 32:0, PlsCho 34:1, and PlsCho 36:0, whereas the variables belonging to PlsEtn containing more unsaturated fatty acyl (such as PlsEtn 36:5, PlsEtn 36:6, PlsEtn 40:7, and PlsEtn 38:4) orientated to the negative *x*-axis, suggesting that porcine brain products were enriched with the unsaturated plasmalogen species compared with others.

When focusing on the plasmalogen profiles obtained through different processing techniques (Figure 4D,E), we observed that the spots of Products 1 and 2 clearly clustered in the upper-right part of the score plot and were loosely distributed, distinct from the other samples. This suggested that although there was no significant difference in the total amount of plasmalogens between the raw products and the glycerophospholipid products, there were compositional differences in terms of the species level. Furthermore, among those processed products (which were subjected to acetone precipitation and ethanol extraction), Product 4 and Products 3, 5, 6, 7, and 8 also exhibited certain differences in the plasmalogen profile. Based on the extraction process, it could be speculated that the concentration step for the samples during the production process might lead to changes in plasmalogen species; however, the specific biomarkers of these changes and the key factors causing these changes remain unknown. Nevertheless, we will employ techniques such as controlling variables and designing single-factor experiments to investigate this issue in future work.

It should be noted that there were considerable intra-group variations in the plasmalogen profiles of the raw products, which were potentially attributable to batch-to-batch differences or sampling inconsistencies during production. However, the processing of the porcine brain could effectively reduce these distinguishments, thereby enhancing product uniformity and better controlling the quality. Still, some products (e.g., No.4 and No. 7) remained diverse, indicating that further attention should be devoted to more effective homogenization of porcine brain-derived plasmalogen products.

Then, we compared the fatty acyl composition of plasmalogens in the investigated samples (Table 3). For the choline type, the species consisted of FFA18:0 and FFA18:1, in which FFA18:0 was the major fatty acyl. For the ethanolamine type, FFA 18:1 also was the dominant fatty acyl (account for 41.12−55.36%), followed by FFA20:4 (17.78−27.56%), FFA22:6 (13.55−16.10%), and FFA22:4 (3.66−7.31%); the more unsaturated fatty acyl it has, the more beneficial for cardiovascular health.

### 3.5. Nutritional Assessment of Porcine Brain Plasmalogens

For achieving a more comprehensive evaluation of these products, serval nutrient indexes were used to assess the nutrient value by using the fatty acyl composition. All the related indexes and values are listed in Table 4. According to these results, porcine brain-derived products showed more polyunsaturated fatty acids, whereas the egg-derived products were enriched in saturated and monounsaturated fatty acids.

The PUFA/SFA ratio and n-6/n-3 ratio indexes are generally used to assess the impact of diet on cardiovascular health. The higher the PUFA/SFA ratio, the more positive the effect. In terms of the n-6/n-3 ratio, according to the recommendations from the WHO and European Nutritional Societies, an n-6/n-3 value < 5–10 is beneficial for anti-inflammation, as well as improving cardiovascular health and treating neurological disorders [28].

It is widely recognized that, as two well-known n-3 long-chain PUFAs, EPA and DHA in the human body play essential roles in multiple biological processes, such as reducing the risk of cardiovascular diseases, preventing hypertension, and suppressing inflammation. A sufficient EPA + DHA intake is recommended by various dietary guidelines; for instance, a recommended amount of 0.250–2 g/day is recommended by the Food and Agriculture Organization of the United Nations (UN FAO).

## 4. Conclusions

In summary, the current study focused on the plasmalogen determination in porcine brain products. The total plasmalogen amount was high in porcine brain-derived products, followed by egg-derived products, whereas no plasmalogen was detected in soy-derived products. Acetone extraction, ethanol extraction, and drying did not show an apparent effect on the total plasmalogen amount. In terms of plasmalogen profile, for the porcine brain products, industrial processing led to a more stable composition of the molecular species, which differed from the raw products. The nutritional assessment revealed that the porcine brain-derived plasmalogens could be considered a healthy source of functional lipids that benefit cardiovascular health. One of the limitations in the current results was based on a relatively small sample size, which might lack representativeness. Future work should involve an increased number of product batches to obtain more stable findings. Additionally, beyond the two primary types, PlsEtn and PlsCho, there might be more types of plasmalogens derived from other lipids (e.g., phosphatidylglycerol, phosphatidylinositol, or phosphatidic acid) with trace amounts. Future research on analyzing these vinyl-ether-containing phospholipids would help expand the scope of plasmalogen in foods. At the same time, the resolution of the isomers, such as plasmanyl/plasmenyl and isomers with the same CN/DB, should be improved with the help of a better chromatographic separation or ion mobility spectrometry. Moreover, the composition of porcine brain products is actually quite complex, and the evaluation of product quality should not rely solely on plasmalogen content or composition. Therefore, it is necessary to incorporate multiple nutritional components, such as amino acids and fatty acids, to establish a more comprehensive quality assessment system. Our study not only shows the feasibility of producing porcine brain-derived plasmalogens, but also provides possible strategies for developing and quality-controlling dietary plasmalogen supplements and healthcare products.

## Figures and Tables

**Table 1:** Characteristics and organic solvent residue of porcine brain products.

Index	No. 1	No. 2	No. 3	No. 4	No. 5	No. 6	No. 7	No. 8
Acetone residue (%)	ND	ND	0.001	0.003	0.001	0.003	0.002	0.002
Ethanol residue (%)	ND	ND	0.005	0.005	0.005	0.020	0.010	0.010

ND, not detected.

**Table 2:** Identification of plasmalogen species in porcine brain tissue samples.

Plasmalogen Species	LIMIDMAPS Nomenclature		RT (min)	High-Resolution MS1 Signals				MS/MS Signals
	Species Level	Molecular Species Level		Ion	Calc. *m*/*z*	Exp. *m*/*z*	Δppm	
PlsEtn								
PlsEtn30:0	PE P-30:0	NA	10.71	[M−H]^−^	646.4817	646.4823	−0.93	ND
PlsEtn32:0	PE P-32:0	NA	11.08	[M−H]^−^	674.5130	674.5143	−1.93	ND
PlsEtn32:1	PE P-32:1	PE P-16:0/16:1	10.77	[M−H]^−^	672.4974	672.4985	−1.64	253,436,418
PlsEtn32:2	PE P-32:2	NA	10.54	[M−H]^−^	670.4817	670.4827	−1.49	ND
PlsEtn33:0	PE P-33:0	PE P-16:0/17:0 (ISTD)	11.25	[M−H]^−^	688.5287	688.5300	−1.89	269,436,418
PlsEtn34:0	PE P-34:0	NA	11.51	[M−H]^−^	702.5443	702.5458	−2.14	ND
PlsEtn34:1	PE P-34:1	PE P-16:0/18:1	11.14	[M−H]^−^	700.5287	700.5306	−2.71	281,436,418
PlsEtn34:2	PE P-34:2	PE P-16:0/18:2	10.85	[M−H]^−^	698.5130	698.5153	−3.29	279,436,418
PlsEtn34:3	PE P-34:3	NA	10.60	[M−H]^−^	696.4974	696.4990	−2.30	ND
PlsEtn34:4	PE P-34:4	NA	10.54	[M−H]^−^	694.4817	694.4812	0.72	ND
PlsEtn36:1	PE P-36:1	PE P-18:0/18:1	11.59	[M−H]^−^	728.5600	728.5611	−1.51	281,464,446
PlsEtn36:2	PE P-36:2	PE P-18:1/18:1	11.22	[M−H]^−^	726.5443	726.5464	−2.89	281,462,444
PlsEtn36:3	PE P-36:3	PE P-16:0/20:3 PE P-18:1/18:2	11.11	[M−H]^−^	724.5287	724.531	−3.17	305,436,418 279,462,444
PlsEtn36:4	PE P-36:4	PE P-16:0/20:4	10.88	[M−H]^−^	722.5130	722.5130	0.00	303,436,418
PlsEtn36:5	PE P-36:5	PE P-16:0/20:5	10.54	[M−H]^−^	720.4974	720.4975	−0.14	301,436,418
PlsEtn36:6	PE P-36:6	NA	10.54	[M−H]^−^	718.4817	718.4816	0.14	ND
PlsEtn38:4	PE P-38:4	PE P-18:0/20:4 PE P-16:0/22:4	11.30	[M−H]^−^	750.5443	750.5421	2.93	303,464,446 331,436,418
PlsEtn38:5	PE P-38:5	PE P-18:1/20:4	10.96	[M−H]^−^	748.5287	748.5265	2.94	303,462,444
PlsEtn38:6	PE P-38:6	PE P-18:2/20:4 PE P-16:0/22:6	10.85	[M−H]^−^	746.5130	746.5110	2.68	303,460,442 327,436,418
PlsEtn38:7	PE P-38:7	NA	10.60	[M−H]^−^	744.4974	744.4978	−0.54	ND
PlsEtn40:6	PE P-40:6	PE P-18:0/22:6	11.25	[M−H]^−^	774.5443	774.5421	2.84	327,464,446
PlsEtn40:7	PE P-40:7	PE P-18:1/22:6	10.91	[M−H]^−^	772.5287	772.5281	0.78	327,462,444
PlsEtn40:8	PE P-40:8	NA	10.71	[M−H]^−^	770.5130	770.5124	0.78	ND
PlsCho								
PlsCho33:0	PC P-33:0	PC P-16:0/17:0 (ISTD)	11.01	[M+CH_3_COO]^−^	790.5967	790.5959	1.01	269,464,716
PlsCho34:0	PC P-34:0	PC P-16:0/18:0	11.28	[M+CH_3_COO]^−^	804.6124	804.6118	0.75	283,464,730
PlsCho34:1	PC P-34:1	PC P-16:0/18:1	11.22	[M+CH_3_COO]^−^	802.5967	802.5958	1.12	281,464,728
PlsCho36:0	PC P-36:0	PC P-18:0/18:0	11.85	[M+CH_3_COO]^−^	832.6437	832.6443	−0.72	283,492,758
PlsCho36:1	PC P-36:1	PC P-18:0/18:1	11.48	[M+CH_3_COO]^−^	830.6280	830.6282	−0.24	281,492,756

NA, not available; identification of molecular species requires MS/MS data. ND, not detected.

**Table 3:** Fatty acyl composition (%) in the porcine brain tissue samples.

	Sample Code																	
	1		2		3		4		5		6		7		8		9	
	Etn	Cho	Etn	Cho	Etn	Cho	Etn	Cho	Etn	Cho	Etn	Cho	Etn	Cho	Etn	Cho	Etn	Cho
16:1	2.01	ND	2.40	ND	1.83	ND	1.31	ND	1.95	ND	1.51	ND	1.53	ND	1.36	ND	0.29	ND
18:0	ND	76.07	ND	77.86	ND	80.97	ND	75.30	ND	81.50	ND	79.31	ND	80.57	ND	78.28	ND	44.64
18:1	45.45	23.93	41.12	22.14	45.66	19.03	44.13	24.70	45.95	18.50	45.39	20.69	44.80	19.43	45.69	21.72	56.06	55.36
18:2	3.05	ND	3.31	ND	2.98	ND	2.76	ND	2.91	ND	2.82	ND	2.83	ND	2.76	ND	2.92	ND
20:3	0.62	ND	0.63	ND	0.60	ND	0.64	ND	0.58	ND	0.63	ND	0.61	ND	0.61	ND	0.76	ND
20:4	27.22	ND	27.56	ND	25.29	ND	26.49	ND	24.79	ND	25.51	ND	25.76	ND	25.29	ND	17.78	ND
20:5	0.32	ND	0.31	ND	0.36	ND	0.22	ND	0.37	ND	0.26	ND	0.28	ND	0.24	ND	ND	ND
22:4	6.46	ND	6.66	ND	6.92	ND	7.24	ND	6.90	ND	7.11	ND	7.15	ND	7.31	ND	3.66	ND
22:6	13.90	ND	16.93	ND	15.06	ND	16.10	ND	15.27	ND	15.63	ND	15.91	ND	15.70	ND	13.55	ND
Others	0.97	ND	1.08	ND	1.30	ND	1.11	ND	1.29	ND	1.13	ND	1.12	ND	1.03	ND	4.97	ND

Etn, ethanolamine; Cho, choline. ND, not detected.

**Table 4:** Nutrient index of fatty acids in plasmalogens from the investigated samples.

Index	Sample Code								
	1	2	3	4	5	6	7	8	9
Σ_SFA_	0.017 ± 0.001	0.015 ± 0.001	0.017 ± 0.002	0.033 ± 0.002	0.016 ± 0.002	0.016 ± 0.004	0.018 ± 0.002	0.018 ± 0.002	0.037 ± 0.005
Σ_MUFA_	0.469 ± 0.029	0.431 ± 0.001	0.469 ± 0.004	0.445 ± 0.002	0.473 ± 0.003	0.464 ± 0.009	0.457 ± 0.003	0.465 ± 0.010	0.563 ± 0.003
Σ_PUFA_	0.504 ± 0.028	0.543 ± 0.002	0.502 ± 0.003	0.511 ± 0.002	0.498 ± 0.002	0.509 ± 0.008	0.514 ± 0.005	0.507 ± 0.011	0.355 ± 0.006
Σ_PUFA_/Σ_SFA_	30.0 ± 1.6	35.8 ± 2.6	30.7 ± 3.5	15.6 ± 1.1	31.9 ± 4.0	32.4 ± 6.6	28.3 ± 2.7	27.7 ± 3.3	9.7 ± 1.4
n-6/n-3	2.58 ± 0.10	2.18 ± 0.02	2.28 ± 0.02	2.24 ± 0.06	2.21 ± 0.01	2.23 ± 0.02	2.21 ± 0.01	2.22 ± 0.01	1.80 ± 0.04
IT	0.014 ± 0.001	0.011 ± 0.001	0.013 ± 0.002	0.025 ± 0.002	0.012 ± 0.001	0.012 ± 0.003	0.014 ± 0.001	0.014 ± 0.001	0.028 ± 0.004
UI	270 ± 9	287 ± 1	271 ± 1	274 ± 1	271 ± 0	275 ± 3	276 ± 2	274 ± 4	217 ± 3
EPA + DHA	18.2 ± 2.0	13.8 ± 1.2	22.2 ± 1.5	21.9 ± 1.7	24.5 ± 0.9	24.3 ± 1.6	25.0 ± 2.0	27.7 ± 1.1	0.4 ± 0.0

SFA, saturated fatty acids; MUFA, monounsaturated fatty acids; PUFA, polyunsaturated fatty acids; TI, thrombogenicity index; UI, unsaturation index; EPA, eicosapentaenoic acid; DHA, docosahexaenoic acid.

## Data Availability

Data are contained within the article or Supplementary Materials.

## Supplementary Materials

The following supporting information can be downloaded at: https://www.mdpi.com/article/10.3390/foods12162990/s1, Table S1: Sample information.
