# Supplementary material for: Plasmalogen Profiling in Porcine Brain Tissues by LC-MS/MS
Source: Foods. 2023 Aug 8;12(16):2990. doi: 10.3390/foods12162990 (PMC10453910; doi:10.3390/foods12162990)
Supplement: Supplementary file 1 [file foods-12-02990-s001.zip › foods-2467055-supplementary.pdf]

Plasmalogen profiling in porcine brain tissues by LC-MS/MS

Supporting Information

Table S1. Sample information

Sample code	Sample type	Sample source	Sample description
1	Powder	Porcine brain	Raw tissue<br>(fresh, freeze-dried)
2	Powder	Porcine brain	Raw tissue<br>(stored at −20°C for 6 months, freeze-dried)
3	Powder	Porcine brain	Purified glycerophospholipid product<br>(spray-dried)
4	Powder	Porcine brain	Purified glycerophospholipid product<br>(freeze-dried)
5	Powder	Porcine brain	Purified glycerophospholipid product<br>(freeze-dried)
6	Powder	Porcine brain	Purified glycerophospholipid product<br>(freeze-dried)
7	Powder	Porcine brain	Purified glycerophospholipid product<br>(freeze-dried)
8	Powder	Porcine brain	Purified glycerophospholipid product<br>(freeze-dried)
9	Powder	Egg	Commercial lecithin product
10	Powder	Soy	Commercial lecithin product
11	Powder	Soy	Commercial lecithin product
12	Powder	Soy	Commercial lecithin product
